# Reduced Resting-State EEG Power Spectra and Functional Connectivity after 24 and 36 Hours of Sleep Deprivation

**DOI:** 10.3390/brainsci13060949

**Published:** 2023-06-14

**Authors:** Jie Lian, Lin Xu, Tao Song, Ziyi Peng, Zheyuan Zhang, Xin An, Shufang Chen, Xiao Zhong, Yongcong Shao

**Affiliations:** School of Psychology, Beijing Sport University, Beijing 100084, China; lianjie0722@163.com (J.L.); rzxulin1997@126.com (L.X.); songtaozy@163.com (T.S.); pzyi121@163.com (Z.P.); zoey_zhang2121@163.com (Z.Z.); axinlll@163.com (X.A.); chenshufang0617@163.com (S.C.); zhongx2022@bsu.edu.cn (X.Z.)

**Keywords:** sleep deprivation, resting state, source localization analysis, power spectrum analysis, functional connectivity

## Abstract

Total sleep deprivation (TSD) leads to cognitive decline; however, the neurophysiological mechanisms underlying resting-state electroencephalogram (EEG) changes after TSD remain unclear. In this study, 42 healthy adult participants were subjected to 36 h of sleep deprivation (36 h TSD), and resting-state EEG data were recorded at baseline, after 24 h of sleep deprivation (24 h TSD), and after 36 h TSD. The analysis of resting-state EEG at baseline, after 24 h TSD, and after 36 h TSD using source localization analysis, power spectrum analysis, and functional connectivity analysis revealed a decrease in alpha-band power and a significant increase in delta-band power after TSD and impaired functional connectivity in the default mode network, precuneus, and inferior parietal lobule. The cortical activities of the precuneus, inferior parietal lobule, and superior parietal lobule were significantly reduced, but no difference was found between the 24 h and 36 h TSD groups. This may indicate that TSD caused some damage to the participants, but this damage temporarily slowed during the 24 h to 36 h TSD period.

## 1. Introduction

Total sleep deprivation (TSD) is a state of continuous sleep deprivation for at least 24 h [1], and is now commonplace, especially among professionals such as nurses, doctors, police officers, and military personnel. TSD has a significant negative impact on daily life, significantly increasing the risk of accidents and affecting a range of cognitive functions, including alertness [2], sustained attention [3], working memory [4], inhibitory control [5], emotions [6], and decision-making [7]. In addition, recent evidence suggests that TSD affects social decision making [8]. It has also been estimated by magnetic resonance imaging that TSD produces changes in the brain similar to one or two years of aging [9]. Thus, TSD has significant detrimental effects on both individuals and society.

Electroencephalography (EEG) is a sensitive tool that can be used to assess the cognitive processes underlying vigilance. EEG is commonly used to understand the effects of TSD on the neurophysiological activity of the brain and the function of brain areas during the waking state of an individual. EEG is currently used as a neurophysiological tool to study brain network changes following TSD. The following EEG frequency classifications are widely used: Delta (1–4 Hz), Theta (4–8 Hz), Alpha (8–13 Hz), Beta (13–30 Hz), and Gamma (30–50 Hz). EEG spectral analysis has demonstrated that prolonged TSD can alter cortical activity in the brain [10,11].

Alpha EEG power is commonly used to study the effects of TSD. It has been shown that alpha power gradually decreases and theta power increases during the transition from a resting state with eyes closed to sleep [12,13,14]. If drowsiness becomes more intense in participants as sleep deprivation progresses, a decrease in alpha power may be observed. Thus, a decrease in alpha power during wakefulness may indicate a strong motivation for sleep. In addition, the early stages of drowsiness after prolonged wakefulness are characterized by “alpha power dropout”, in which the slowing of alpha activity is gradually replaced by theta and delta activity as sleep motivation increases.

In addition to alpha power in the EEG, studies have revealed a significant increase in delta and theta power activity after TSD [15]. EEG results have revealed an increase in low-frequency band power with increasing wakefulness, which is considered the main EEG marker of drowsiness and is particularly evident in the central frontal region [16,17,18]. Ferreira (2006) [19] collected data from 11 healthy young participants after one night of TSD and found an increase in theta and delta relative power in the occipital and temporal lobes, a decrease in beta absolute power in the temporal regions, and a decrease in alpha absolute power centered in the frontal and temporal lobes. De Gennaro (2007) [16] used power analysis before and after 40 h of TSD (40 h TSD) in 33 healthy participants and found that TSD resulted in a severe increase in subjective drowsiness, with a dramatic decrease in alertness. EEG topography revealed a substantial increase in delta and theta activity, which was more pronounced in the central frontal region. Miraglia (2021) [20] assessed the effects of TSD using functional connectivity (FC) analysis and found changes in brain connectivity after 40 h of TSD, with participants exhibiting significant changes in the small-world metric; that is, the lower frequency bands (delta and theta bands) decreased and the higher frequency bands increased in power. In non-rapid eye movement sleep and rapid eye movement sleep, EEG power increased throughout the low-frequency (<8 Hz) range, whereas activity decreased in the higher-frequency range (12–15 Hz) [21,22,23].

Other studies have used graph theory and other methods to investigate changes in the FC of brain networks after TSD. Verweij (2014) [24] showed that 24 h of TSD (24 h TSD) significantly affected the FC of the prefrontal regions. Graph theory revealed a decrease in the clustering coefficient in the alpha band and an increase in the path length in the theta band, suggesting a possible problem in prefrontal function. Kar (2011) [25] used graph theory to analyze FC after 36 h of TSD (36 h TSD). Increased fatigue and sleepiness were associated with increased connectivity, clustering coefficient, small-world metric, and sleepiness. Koenis (2013) [26] showed that after 24 h TSD, the network was more random in the alpha band with the eyes closed, as demonstrated by a decrease in clustering coefficients and characteristic path lengths. However, in the gamma band, a more ordered network was found in the eyes-open state, which was consistent with an increase in clustering coefficients and characteristic path lengths. TSD reduced alpha power with eyes open and closed and increased power at frequencies below 8 Hz with eyes closed.

Numerous functional magnetic resonance imaging (fMRI) studies have investigated how TSD affects the activity of various brain regions. These findings imply that TSD may have an impact on several brain regions, resulting in imbalances in total network integration and changing the connectivity and functionality of regions such the frontoparietal network and the default mode network (DMN) [27,28]. TSD lowers FC within the DMN and between the DMN and the anti-correlated network, according to studies using resting-state functional connectivity fMRI analysis [29,30], indicating that changes in functional brain connectivity are a result of TSD. Liu (2014) [31] found significant enhancement in the small-world network after 34 h of TSD (34 h TSD) by calculating clustering coefficients and path lengths, suggesting that compensatory adaptation may exist in the human brain. The lower degree of inhibition of negative FC under sleep deprivation conditions may indicate selective sensitivity to sleep deprivation in regions such as the occipital, sensorimotor, and prefrontal-parietal lobes, where there is an overall decrease in inhibition. In sleep deprivation studies, researchers have explored changes in network connectivity after sleep deprivation using fMRI techniques, but few EEG recordings have been made to measure changes in connectivity between brain regions.

Although the harmful effects of TSD on individual cognitive function have been confirmed in numerous studies, some have found that different durations of sleep deprivation may have different effects on individual cognitive function [32]. At the behavioral level, Stenson (2023) [33] subjected 12 adults to 36 h of TSD and found that attentional functioning tended to decline between 20 and 36 h of TSD. Skurvydas (2021) [32] showed no significant change in reaction time to complete the Go/No-Go task after 24 h of TSD in 19 healthy adult males, but a significant increase in reaction time to the Go/No-Go task after 48 h of TSD (48 h TSD). Chua (2017) [34] administered 18 attention tests to 30 healthy males during 40 h TSD and found a decreasing trend in individual performance between 16 and 40 h of TSD.

In addition, studies have explored the effects of different sleep deprivation durations in terms of time course and spatial characteristics through event-related potentials (ERP) and fMRI techniques. In terms of EEG, Zhang (2021) [35] subjected 64 healthy male college students to 30 h of TSD (30 h TSD) and found that the accuracy of working memory tasks was significantly lower after 30 h of TSD compared to 24 h TSD, and induced larger N200 difference waves. Gosselin (2019) [36] measured six Go/No-Go tasks and found a significant increase in error rates after 24 and 36 h of TSD and a significant decrease in NoGo-P3 wave amplitude after 24 and 36 h of TSD. In terms of fMRI, Chee (2006) [37] assessed working memory in 26 healthy young adults and found significantly lower working memory task performances after 24 and 35 h of TSD compared to baseline, but no significant differences between task performance following 24 and 35 h of TSD. Task-related activation was reduced in both the supraparietal areas and the left thalamus in both post-sleep deprivation measures.

In summary, few studies have investigated the effects of sleep deprivation for different durations on brain activation and FC in the resting state; therefore, an in-depth analysis of resting state EEG after sleep deprivation is needed. In particular, changes in resting-state EEG after different durations of sleep deprivation are not well defined. Therefore, this study investigated the effect of sleep deprivation duration on neural oscillations in different frequency bands of the brain. Although some studies have found power changes in the resting state after TSD, evidence on spatial characteristics is still relatively lacking. The present study further explored the effects of different sleep deprivation durations on the network connectivity of brain regions, using power spectrum, source localization, and functional connectivity analysis. In addition, nowadays sleep problems have become a major health-related issue. This study can provide a way to understand the effects of sleep on cognitive mechanisms in people with insomnia and sleep disorders, and resting-state EEG can collect indicators on individuals in a task-free state. Exploring the effects of different sleep deprivation durations on individual brain mechanisms and their action mechanisms can provide a perspective for psychopathology research and also provide a theoretical basis and guidance for clinical research on dynamic assessment and intervention strategies for patients with insomnia.

In this study, 42 participants were recruited for a 36 h TSD, and EEG data were recorded at baseline, after 24 h TSD, and after 36 h TSD. We performed power spectrum analysis, source localization analysis, and functional connectivity analysis on resting-state EEG data to explore more comprehensively and deeply the differences according to the different durations of sleep deprivation: baseline, 24 h TSD, and 36 h TSD. The hypotheses tested by this study were as follows: (1) low-frequency power decreases and high-frequency power increases after TSD; (2) brain area activation decreases and functional connectivity of the DMN decreases after TSD; and (3) 36 h TSD being more severely impairing than 24 h TSD.

## 2. Materials and Methods

### 2.1. Participants

Sample size estimates were generated with analysis of variance (ANOVA) repeated measures within the design using G*power version 3.1.9.7 [38] (Universitat Kiel, Kiel, Germany). An a priori power analysis was conducted, which indicated that a sample size of 28 participants was required to detect a medium effect (f = 0.25) with a power of 0.80 and a type I error probability of 5%. To account for participant attrition and data culling that could result in an insufficient sample size, 42 participants (22.26 ± 1.68 years, age range: 19–27 years, 15 females) from Beihang University were recruited through poster campaigns. All participants were screened for the following conditions before they could enter the formal experiment: (1) right-handedness, uncorrected or corrected normal vision; (2) no history of alcohol or drug abuse, no history of psychiatric or neurological disorders; (3) all scores on the Pittsburgh Sleep Quality Index test were less than 5, which indicated that all participants had good sleep habits [39]; (4) participants did not have any history of serious physical illness or traumatic brain injury; (5) participants had not taken drugs recently, with no habit of smoking or drinking coffee or tea; and (6) participants were required not to have previously participated in relevant psychophysiological tests.

The experiment was approved by the Ethics Committee of Beihang University and each participant signed an informed consent form prior to participating in the formal experiment. All procedures were performed according to the guidelines of the Declaration of Helsinki.

### 2.2. Experimental Procedures

This study was an experimental design of single-factor repeated measurement. The independent variable was the length of sleep deprivation, including three levels (baseline, 24 h TSD and 36 h TSD). Participants came to the laboratory the night before the formal experiment began and were informed about the experimental procedure and possible risks. Based on previous literature on resting state and sleep [40,41,42], this study collected participants’ eyes-closed resting-state data to ensure that participants were awake. The formal experiment began at 8:00 a.m. the next morning, with resting-state data collection completed with eyes closed for 4 min during the 24 h and 36 h of TSD. All participants avoided alcohol, caffeine, and strenuous exercise on the day before and during the experiment. Participants remained awake throughout the sleep deprivation period and were required to remain inside the experimental setting at all times, accompanied and supervised by a physician and experimenter.

### 2.3. Data Collection and Processing

The experiments were conducted in a soundproof temperature- and humidity-controlled laboratory. While the participants remained awake with their eyes closed, a 10–20 International EEG recording system and a Scan 4.5 (Neuroscan Products) 64-channel EEG recording and analysis system were used to collect EEG signals for 4 min. The participants’ vertical and horizontal EEGs were recorded during the acquisition process, and the bilateral mastoids were used as online reference electrodes. The EEG sampling rate was 1000 Hz, and the impedance of the electrodes was kept below 5 kΩ. Participants completed 4 min of eyes-closed resting-state data acquisition at baseline, after 24 h TSD, and after 36 h TSD. There was no significant difference in the data length (*F* (2, 78) = 0.135, *p* > 0.05, η^2^_p_ = 0.003) or number of interpolated bad leads (*F* (2, 38) = 1.000, *p* > 0.05, η^2^_p_ = 0.05) among the three conditions.

Resting-state data from 42 participants were preprocessed using EEGLAB v21.1.0 [43]. The data were visually browsed, and abnormal EEG data from two participants were excluded. Band-pass filtering (0.1–70 Hz) was performed using the mean reference as a re-reference, and artefacts such as eye artefacts and electromyography were removed using independent components analysis [44]. The sampling rate was reduced to 250 Hz and the data divided into 2 s segments.

### 2.4. Data Analysis

#### 2.4.1. Power Spectral Analysis

Power spectrum analysis of the pre-processed data was performed using a fast Fourier transform (FFT) method. FFT analysis of all EEG data was performed using a 2 s Hanning window. The spectral power was divided into five frequency band ranges: Delta (1–4 Hz), Theta (4–8 Hz), Alpha (8–13 Hz), Beta (13–30 Hz), and Gamma (30–50 Hz). Statistical analysis was performed separately for the three conditions (baseline, 24 h TSD, and 36 h TSD) in the five frequency ranges, using one-way ANOVA of whole-brain power.

#### 2.4.2. Source Localization Analysis

Sloreta software package (version 20201109) [45] was used for source localization. The cross-spectral matrix was calculated for each participant’s data at Delta (1–4 Hz), Theta (4–8 Hz), Alpha (8–13 Hz), Beta (13–30 Hz), and Gamma (30–50 Hz) frequencies as inputs for source localization. The solution space corresponded to 6239 voxels at a spatial resolution of 5 mm × 5 mm × 5 mm. The Montreal Neurological Institute 152 standard template [46] was used as the head model. Statistical analysis of the source localization was performed using statistical nonparametric mapping [47].

#### 2.4.3. EEG Source Connectivity Analysis

Source localization and functional connectivity analyses were performed using FieldTrip [48]. An FFT based on the Hanning window was applied to the data, and source localization was performed using the standard custom-boundary-element method head-volume conduction model in FieldTrip. Spatial filters were computed using partial canonical correlation/coherence as a beamformer method [49]. We divided the brain into 90 regions using the Anatomical Automated Labeling 90 template [50], and then calculated the phase-locking value for the 90 brain regions, which reflects the phase-synchronous relationship between two signals and is commonly used for functional brain connectivity analyses [51]. Finally, we obtained a 40 × 90 × 90 functional connectivity matrix.

The network-based statistics (NBS) method is a nonparametric statistical method used to compare connectivity measures between sets of regions of interest [52]. NBS uses a permutation-based approach to select sub-networks composed of edges with significantly different weights between two groups. These edges must be identified, regardless of whether their weights are strong or weak. One advantage of this technique is that it avoids the problem of multiple comparisons that one faces in the pairwise comparison of all edges between two groups. To improve the statistical power of the analysis, this study used the NBS toolbox to implement a network-based nonparametric statistical method. This method was used to deal with the multiple comparison problems described by Zalesky [52]. This approach has the advantage of exploiting the internal connectivity structure of the brain map in the correction step of multiple comparisons and has the potential to provide substantial statistical power. The statistical inference of NBS is similar to other substitution-based general linear models [53] and has been used in the sleep domain [54]. In this study, we performed 5000 permutations with very strict threshold levels (*p* < 0.0001) to detect differences. GraphPad Prism v8.3.0 and circos v0.69–6 [55] were used to achieve visualization.

## 3. Results

### 3.1. Power Spectrum Results

**Figure 1:**
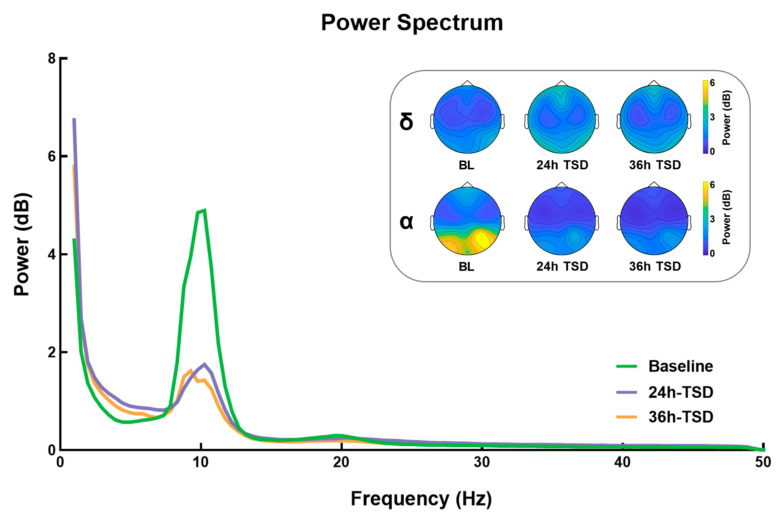
Differences between power and topographic maps under three conditions: baseline (BL), 24 h of sleep deprivation (24 h TSD), and 36 h of sleep deprivation (36 h TSD).

As shown in Figure 1, compared to baseline, the 24 h and 36 h TSD data had significantly higher power in the delta band (*F* (2, 78) = 9.988, *p* < 0.001, $\eta_{p}^{2}$ = 0.204) and significantly lower power in the alpha band (*F* (2, 38) = 14.238, *p* < 0.001, $\eta_{p}^{2}$ = 0.428), but no significant differences were found between the 24 h and 36 h TSD. No significant differences were observed in other frequency bands (*p* > 0.05).

### 3.2. Source Location Results

**Figure 2:**
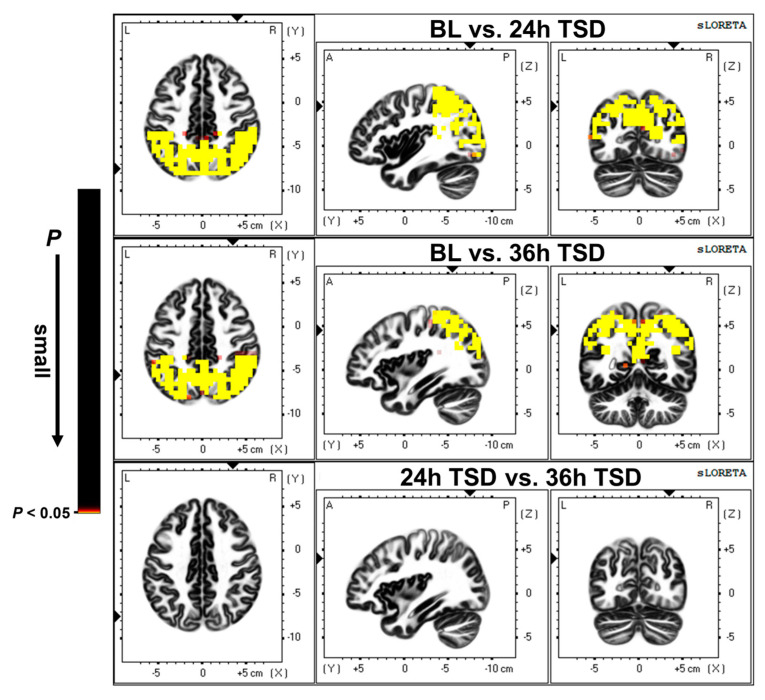
Differences in source activation between baseline (BL) versus (vs.) 24 h of sleep deprivation (24 h TSD), BL vs. 36 h of sleep deprivation (36 h TSD), and 24 h TSD vs. 36 h TSD. The significance level was set at *p* < 0.05.

As shown in Figure 2, compared with the baseline, 24 h TSD was followed by a broad reduction in cortical activity, primarily in the precuneus (PrC), inferior parietal lobule (IPL), superior parietal lobule, and postcentral gyrus (*t* = 0.871, *p* < 0.01).

After 36 h of TSD, there was a widespread reduction in cortical activity compared to baseline, with significant deactivation in the PrC, IPL, superior parietal lobule, and cingulate gyrus (*t* = 1.024, *p* < 0.01).

### 3.3. EEG Source Connectivity Results

Significant functional connectivity was found only in the alpha band (8–13 Hz). Compared with the baseline, functional connectivity was significantly reduced after 24 h of TSD, primarily in the DMN, attention network, and visual network, involving the superior occipital gyrus, superior parietal gyrus, and middle temporal gyrus (*t* = 4.105, *p* < 0.0001).

**Figure 3:**
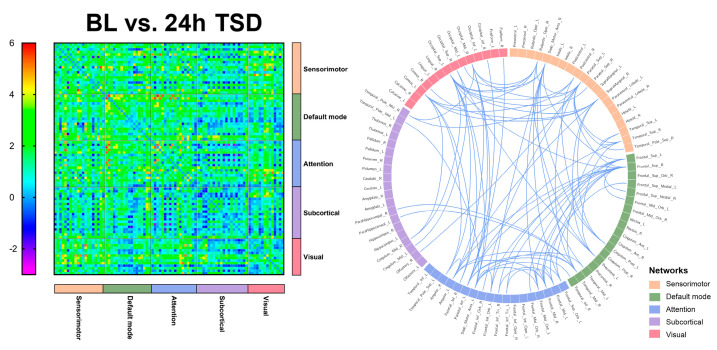
Functional connectivity between baseline (BL) versus (vs.) 24 h of sleep deprivation (24 h TSD) in 90 brain regions, and significant functional connectivity among brain regions.

**Figure 4:**
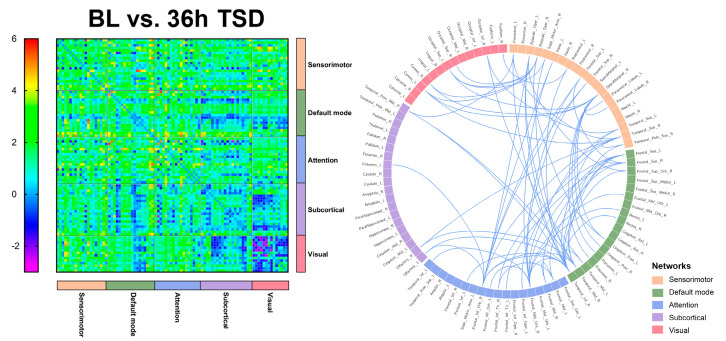
Functional connectivity between baseline (BL) versus (vs.) 36 h of sleep deprivation (36 h TSD) in 90 brain regions, and significant functional connectivity among brain regions.

Significantly lower functional connectivity after 36 h TSD was observed compared to baseline, primarily in the DMN, visual network, and sensorimotor network, involving the middle temporal gyrus, superior parietal gyrus, superior temporal gyrus, inferior temporal gyrus, and supramarginal gyrus (*t* = 4.105, *p* < 0.0001). The results are shown in Figure 3 and Figure 4 (See Supplementary Materials for details).

## 4. Discussion

In this study, EEG was recorded under three conditions: baseline, after 24 h TSD, and after 36 h TSD. The effects of different sleep deprivation durations in each frequency band in the resting state were clarified using power spectrum analysis, source localization analysis, and functional connectivity analysis. The results of this study showed that compared to baseline, power in the alpha band decreased and the power in the delta band increased after 24 and 36 h of TSD. In addition, reduced activation in brain regions was found, especially in the PrC, inferior parietal, and superior parietal regions, after 24 and 36 h of TSD, and FC between the DMN and visual and sensorimotor networks was also significantly reduced after TSD. However, there was no significant difference between 24 h and 36 h of TSD.

The present study found that the power of different frequency bands exhibited different changes after TSD. Power in the alpha band decreased significantly after 24 and 36 h of TSD, whereas power in the delta band increased significantly. It has been shown that, as the process of sleep deprivation deepens, participants become increasingly subjectively sleepy and motivated to sleep, and a decrease in alpha power during wakefulness may be observed [10]. In contrast, the power results in the low-frequency band were opposite to those in the high-frequency band, power in the delta band was significantly higher after TSD, and the topography showed that the effects were primarily concentrated in the frontal regions. In Tinguely (2006) [18], power spectral analysis of the EEG data of eight healthy male adults subjected to 40 h TSD revealed an increase in EEG power after TSD in the low-frequency range (1–8 Hz). Overall, EEG power in the low-frequency range tends to decrease after prolonged wakefulness and always exhibits a maximum in the fronto-centric region [17,18]. This suggests that TSD may have negative effects on higher-order (frontal) cortical areas, and that frontal areas are highly vulnerable to sleep deprivation [56].

In the present study, source localization analysis revealed that brain regions with reduced activation after TSD were predominately concentrated in the PrC, IPL, superior parietal lobule, postcentral gyrus, and cingulate gyrus, which are often reported as brain regions active after sleep deprivation in fMRI studies [57]. Studies have found disturbed activity in the DMN during wakefulness in disorders related to sleep abnormalities, such as schizophrenia and anxiety disorders, which may indicate that the DMN plays a regulatory role in sleep [58]. Anatomically, the DMN spans the bilateral IPL, posterior cingulate cortex, PrC, medial prefrontal cortex, retrosplenial cortex, parts of the hippocampal formation, and medial temporal lobe [59]. The PrC and cingulate cortex are central to the default mode network, are involved in cognitive processes in the brain, and play important roles in regulating DMN activity [60]. Studies have found that disorders such as obstructive sleep apnea and cognitive impairment can lead to impaired PrC, and the adjacent PCC region can be affected to some extent [61]. It has also been shown that cingulate gyrus activity decreases with increasing sleep deprivation duration, which may indicate impaired attention and executive function in individuals with TSD [62]. The superior parietal lobule plays a key role in many sensory and cognitive processes, including spatial perception [63], visuospatial attention [64,65], somatosensory and visuomotor integration [66,67], and reasoning ability [68], among others. Spatial cognitive abilities are fundamental human abilities, and one study found that five days of reduced sleep resulted in reduced visuospatial memory, which may affect an individual’s ability to process images, locate objects in space, and spatially analyze them [69]. Our results showed that the activation of the PrC, cingulate gyrus, postcentral gyrus, and superior parietal lobule was significantly reduced after TSD, which may represent the degree of impairment in executive functions such as spatial cognition, attention, and motor control in individuals after experiencing prolonged sleep deprivation.

The results of the present study indicate that the DMN and attentional and visual networks are significantly affected in individuals after TSD, with significant reductions in FC indicators among networks, especially in the middle temporal, superior temporal, inferior temporal, superior parietal, and superior occipital gyri. It has been found that temporal and parietal activity is reduced during TSD [70]. The temporal lobe is an important brain region responsible for higher cognitive functions that is involved in cognitive processes such as language learning and memory [71]. Verbal learning is a critical cognitive function, and studies have shown that TSD negatively affects verbal learning. Drummond (2000) [56] found that the temporal lobe was significantly less activated in the SD state than in the resting state. Nir (2017) [72] recorded single neuron activity in the medial temporal lobe of neurosurgical patients while they completed a facial recognition test after TSD. The results indicated that cognitive impairment after TSD may be driven by slowed neural activity in the medial temporal lobe of the human brain. An fMRI study based on a nonverbal recognition task showed reduced activation of the inferior temporal gyrus after TSD [73]. Javaheripour (2019) [74] integrated the results of 31 sleep-deprivation-related neuroimaging studies, using an activation likelihood estimation meta-analysis, and found that activity tended to decrease primarily in the right intraparietal sulcus and supraparietal lobules. Federico (2022) [75] examined the effects of self-reported sleep quality on FC in brain regions using fMRI and found that individuals who slept poorly had higher levels of anxiety and depression and poorer cognitive performance, and that sleep quality may modulate FC in limbic and fronto-temporo-parietal brain areas. Participants in another study slept 5 h for 7 consecutive nights [76], and after the first night there were cumulative deficits in both subjective and psychomotor performance measures. Over the week, subjective sleepiness continued to increase and task performance decreased in a linear trend. It follows that individuals experience increased subjective sleepiness [77], more anxiety and depression, and some degree of cognitive impairment during sleep deprivation and that sleep deprivation may lead to impairments in higher cognitive functions, such as language and memory [56].

The DMN has the highest degree of activation in the resting state and plays an important role in the integration of cognitive processes, including those associated with the medial temporal lobe and inferior parietal gyrus. A large-scale network-based study by De Havas (2012) [30] found that the DMN plays a crucial role in attentional processes and that TSD selectively reduces FC within the DMN and its disassociation from anti-correlation networks during the resting state. Ben Simon [78] demonstrated that following a 24 h TSD, the DMN module in the frontal lobe was significantly altered and the executive module network, default mode, salience, and posterior edge of deprivation were all generally lowered. This resulted in decreased task performance and an increase in negative emotions. Kaufmann (2016) [79] showed that 24 h TSD led to significant changes in functional connectivity and demonstrated changes in the DMN, frontal lobe, visual, auditory/language, amygdala/hippocampus, and cerebellar networks after TSD. The results of this study may indicate that after long-term sleep deprivation, individuals’ cognitive functions, such as sustained attention and vigilance and functional connections between networks, are reduced.

In this study, source localization, power spectrum, and functional connectivity analyses were conducted at baseline, after 24 h TSD, and after 36 h TSD. None of the results revealed significant differences between 24 h TSD and 36 h TSD. A previous study documenting the course of 36 h TSD found a significant increase in participants’ error rate in completing tasks after 24 and 36 h of TSD and a sharp decrease in participants’ perceived level of alertness after 18 to 24 h of sustained wakefulness [36]. Drummond (2006) [80] recorded data from 38 healthy adults after 23, 31, and 55 h of TSD and found that error rates were significantly higher after 23, 31, and 55 h of TSD; however, the differences among the three deprivation conditions were not significant. Chee (2006) [37] assessed working memory during 24 and 35 h of TSD in 26 healthy young adults, and although it was found that at 35 h of TSD there was greater variability in performance, there was no significant difference in the degree of decline in performance during the two TSD tests. The process of sleep deprivation in the resting state has also been explored by Li (2020) [81], who performed 28 and 52 h of TSD and used a sliding-window correlation approach to investigate changes in FC over time after TSD. The study found that 52 h of TSD induced a higher frequency of state transitions, suggesting that prolonged wakefulness accelerates transitions between different connection states for all participants. However, this effect was not observed after 28 h of TSD treatment. Alonso (2016) [82] recorded EEG during 36 h TSD in 18 healthy adults and found that although transfer entropy significantly decreased after 36 h TSD, the decrease in information transfer during sleep deprivation predominately started after 24 h of TSD.

The present study found that although neural activity in the brain was impaired after sleep deprivation, no significant differences in neural oscillations were found between 24 and 36 h TSD. Hence, we hypothesize that the harm to individuals from sleep deprivation has already occurred after 24 h TSD, while between 24 and 36 h after sleep deprivation participants are engaged in an adaptation process and there may be a “plateau period.” This may indicate that the cognitive impairment of individuals during short-term TSD does not tend to decline linearly, but temporarily slows at some point; in contrast, as the duration of sleep deprivation increases, certain compensatory mechanisms may emerge in participants’ brain regions to maintain the normal activity and alertness of individuals, among other behavioral effects. This result suggests that when facing the harm caused by sleep deprivation, effective interventions can be carried out at 24 and 36 h of sleep deprivation to prevent further aggravation of damage. The participants in this study were young adults, and previous studies have shown reduced sleep spindle wave duration, peak and mean amplitude in older adults relative to younger adults, predicting that compared to older adults, young adults [83,84] have a better recovery of hippocampal encoding activity and situational learning ability. In contrast, a common feature of age-related cognitive decline is an impaired ability to form new hippocampal-dependent memories [85,86]. Lim [87] observed a relationship between shortened sleep duration and reduced lateral prefrontal gray matter volume in older adults, and further predicted the effects of shortened sleep duration severity. Thus, age may be a factor influencing the effects of sleep deprivation, and the exploration of the effects of sleep deprivation on brain mechanisms at different ages could be developed in future studies.

Our study has some limitations, despite the fact that it adds to the body of research in this area. The present study first investigated resting-state EEG changes at baseline, 24, and 36 h after TSD during sleep deprivation. However, further research is still required to determine how long people can go without sleeping. Second, the study’s participant sex ratio was unequal, and therefore conclusions should not be extrapolated due to this factor alone.

## 5. Conclusions

This study measured resting-state EEG data at baseline, 24 h, and 36 h of TSD, and explored the differences between neural oscillations after TSD and across sleep deprivation durations using source localization, power spectrum, and functional connectivity analyses. After TSD, we observed a decrease in alpha power and an increase in delta power; a decrease in the activation of the PrC, superior parietal lobule, and IPL; and a decrease in the functional connectivity of the DMN. However, no difference was found between 24 and 36 h of TSD, which may indicate that TSD impairs cognitive functions such as alertness and attention. However, this impairment did not have a linear downward trend, and may temporarily slow during 24 to 36 h of TSD.

## Data Availability

The datasets generated for this study are available on request to corresponding authors.

## Supplementary Materials

The following supporting information can be downloaded at: https://www.mdpi.com/article/10.3390/brainsci13060949/s1, Table S1: 81 pairs of significant functionally connected brain regions and t values for baseline and 24 h of sleep deprivation; Table S2: 69 pairs of significant functionally connected brain regions and t values for baseline and 36 h of sleep deprivation.
