# Supplementary material for: Reduced Resting-State EEG Power Spectra and Functional Connectivity after 24 and 36 Hours of Sleep Deprivation
Source: Brain Sci. 2023 Jun 14;13(6):949. doi: 10.3390/brainsci13060949 (PMC10296734; doi:10.3390/brainsci13060949)
Supplement: Supplementary file 1 [file brainsci-13-00949-s001.zip › brainsci-2420894-supplementary.pdf]

Table S1. 81 pairs of significant functionally connected brain regions and t values for baseline and 24 hours of sleep deprivation.

Number	Brain region one	Brain region two	*t*
1	Frontal_Sup_L	Frontal_Sup_R	5.689
2	Frontal_Sup_R	Frontal_Sup_Orb_L	4.827
3	Frontal_Sup_R	Frontal_Mid_L	5.535
4	Frontal_Sup_L	Frontal_Mid_R	5.741
5	Frontal_Mid_L	Frontal_Mid_R	5.404
6	Frontal_Sup_R	Frontal_Mid_Orb_L	5.300
7	Frontal_Mid_R	Frontal_Mid_Orb_L	4.440
8	Frontal_Sup_L	Frontal_Mid_Orb_R	5.462
9	Frontal_Sup_R	Frontal_Inf_Tri_L	5.387
10	Frontal_Mid_R	Frontal_Inf_Tri_L	4.912
11	Frontal_Sup_L	Frontal_Inf_Tri_R	5.333
12	Frontal_Mid_L	Frontal_Inf_Tri_R	4.959
13	Frontal_Sup_R	Frontal_Inf_Orb_L	4.976
14	Frontal_Sup_Orb_L	Supp_Motor_Area_L	4.504
15	Frontal_Mid_Orb_L	Supp_Motor_Area_L	4.906
16	Frontal_Inf_Orb_L	Supp_Motor_Area_L	4.426
17	Frontal_Sup_L	Supp_Motor_Area_R	4.229
18	Frontal_Sup_Orb_L	Supp_Motor_Area_R	5.001
19	Frontal_Mid_Orb_L	Supp_Motor_Area_R	5.472
20	Frontal_Inf_Orb_L	Supp_Motor_Area_R	4.729
21	Frontal_Mid_R	Frontal_Sup_Medial_L	4.316
22	Frontal_Sup_L	Frontal_Sup_Medial_R	4.619
23	Frontal_Sup_R	Frontal_Mid_Orb_L	4.347
24	Supp_Motor_Area_R	Cingulum_Post_L	4.445
25	Rolandic_Oper_R	Cuneus_L	4.469
26	Cingulum_Mid_R	Cuneus_L	4.433
27	Frontal_Sup_R	Occipital_Sup_L	4.409
28	Rolandic_Oper_R	Occipital_Sup_L	4.650
29	Supp_Motor_Area_L	Occipital_Sup_L	4.196
30	Supp_Motor_Area_R	Occipital_Sup_L	4.152
31	Cingulum_Mid_R	Occipital_Sup_L	4.680
32	Cingulum_Mid_L	Occipital_Sup_R	4.159
33	Supp_Motor_Area_R	Occipital_Mid_L	4.407
34	Rolandic_Oper_R	Occipital_Mid_R	4.165
35	Supp_Motor_Area_L	Occipital_Mid_R	4.567
36	Cingulum_Post_L	Postcentral_R	4.458
37	Cuneus_L	Postcentral_R	4.390
38	Occipital_Sup_L	Postcentral_R	5.000
39	Frontal_Sup_R	Parietal_Sup_L	4.451
40	Frontal_Inf_Orb_R	Parietal_Sup_L	4.353

41	Rolandic_Oper_R	Parietal_Sup_L	4.563
42	Olfactory_R	Parietal_Sup_L	4.212
43	Occipital_Mid_R	Parietal_Sup_L	4.782
44	Occipital_Sup_L	Parietal_Sup_R	5.105
45	Frontal_Sup_R	Parietal_Inf_L	4.340
46	Occipital_Mid_R	Parietal_Inf_L	5.297
47	Occipital_Inf_R	Parietal_Inf_L	4.183
48	Fusiform_R	Parietal_Inf_L	4.634
49	Cuneus_L	Parietal_Inf_R	4.747
50	Occipital_Sup_L	Parietal_Inf_R	5.232
51	Cingulum_Post_L	SupraMarginal_R	4.564
52	Cuneus_L	SupraMarginal_R	5.255
53	Cuneus_R	SupraMarginal_R	4.127
54	Occipital_Sup_L	SupraMarginal_R	5.197
55	Frontal_Sup_R	Angular_L	4.682
56	Frontal_Mid_R	Angular_L	4.113
57	Occipital_Mid_R	Angular_L	4.171
58	SupraMarginal_R	Angular_L	4.147
59	Cuneus_L	Angular_R	4.484
60	Occipital_Sup_L	Angular_R	4.592
61	Angular_L	Angular_R	4.408
62	Rolandic_Oper_R	Precuneus_L	4.420
63	Occipital_Sup_R	Precuneus_L	4.467
64	SupraMarginal_R	Precuneus_L	4.663
65	Parietal_Sup_L	Temporal_Sup_R	5.639
66	Parietal_Inf_L	Temporal_Sup_R	4.694
67	Precuneus_L	Temporal_Sup_R	4.335
68	Temporal_Sup_L	Temporal_Sup_R	4.131
69	Parietal_Sup_L	Temporal_Pole_Sup_R	4.256
70	Supp_Motor_Area_L	Temporal_Mid_L	4.276
71	Supp_Motor_Area_R	Temporal_Mid_L	4.997
72	ParaHippocampal_L	Temporal_Mid_R	4.362
73	ParaHippocampal_R	Temporal_Mid_R	4.236
74	Parietal_Sup_L	Temporal_Mid_R	4.746
75	Parietal_Inf_L	Temporal_Mid_R	4.802
76	Angular_L	Temporal_Mid_R	4.216
77	Temporal_Sup_L	Temporal_Mid_R	4.632
78	Supp_Motor_Area_L	Temporal_Pole_Mid_L	4.485
79	Occipital_Mid_R	Temporal_Pole_Mid_R	4.167
80	Parietal_Sup_L	Temporal_Pole_Mid_R	4.643
81	Parietal_Inf_L	Temporal_Inf_R	4.664

Table S2. 69 pairs of significant functionally connected brain regions and t values for baseline and 36 hours of sleep deprivation.

Number	Brain region one	Brain region two	*t*
1	Frontal_Sup_L	Frontal_Sup_R	4.995
2	Frontal_Sup_R	Frontal_Mid_L	5.268
3	Frontal_Sup_L	Frontal_Mid_R	5.597
4	Frontal_Mid_L	Frontal_Mid_R	4.975
5	Frontal_Sup_L	Frontal_Mid_Orb_R	4.531
6	Frontal_Sup_R	Frontal_Inf_Tri_L	4.313
7	Frontal_Sup_L	Frontal_Inf_Tri_R	4.367
8	Frontal_Mid_L	Frontal_Inf_Tri_R	4.960
9	Frontal_Mid_Orb_R	Frontal_Inf_Orb_L	4.502
10	Frontal_Sup_Orb_R	Rolandic_Oper_L	4.257
11	Frontal_Inf_Orb_R	Rolandic_Oper_L	4.424
12	Frontal_Inf_Orb_R	Olfactory_L	4.731
13	Frontal_Mid_Orb_R	Frontal_Mid_Orb_L	4.824
14	Frontal_Mid_Orb_R	Rectus_L	4.697
15	Frontal_Mid_Orb_R	Insula_L	4.569
16	Frontal_Inf_Orb_R	Insula_L	4.459
17	Rolandic_Oper_R	Cingulum_Post_L	4.320
18	Rolandic_Oper_R	Cuneus_L	4.512
19	Rolandic_Oper_R	Cuneus_R	4.903
20	Rolandic_Oper_R	Occipital_Sup_L	4.136
21	Supp_Motor_Area_R	Occipital_Sup_L	4.611
22	Rolandic_Oper_R	Occipital_Sup_R	4.143
23	Fusiform_R	Postcentral_L	4.495
24	Frontal_Sup_R	Parietal_Sup_L	4.482
25	Frontal_Mid_Orb_R	Parietal_Sup_L	4.360
26	Frontal_Inf_Orb_R	Parietal_Sup_L	4.164
27	Frontal_Mid_Orb_R	Parietal_Sup_R	4.142
28	Frontal_Inf_Orb_R	Parietal_Sup_R	4.133
29	Cingulum_Post_L	SupraMarginal_R	5.666
30	Cingulum_Post_R	SupraMarginal_R	5.105
31	Cuneus_L	SupraMarginal_R	4.753
32	Cuneus_R	SupraMarginal_R	4.351
33	Occipital_Sup_L	SupraMarginal_R	4.582
34	SupraMarginal_R	Precuneus_L	4.224
35	SupraMarginal_R	Precuneus_R	4.213
36	SupraMarginal_R	Paracentral_Lobule_L	4.354
37	SupraMarginal_R	Paracentral_Lobule_R	4.270
38	Frontal_Inf_Orb_R	Putamen_L	4.203
39	Frontal_Mid_Orb_R	Temporal_Sup_L	4.126
40	Occipital_Inf_R	Temporal_Sup_L	4.541

41	Precentral_L	Temporal_Sup_R	4.112
42	Frontal_Inf_Oper_L	Temporal_Sup_R	4.141
43	Frontal_Inf_Orb_R	Temporal_Sup_R	4.340
44	Olfactory_R	Temporal_Sup_R	4.125
45	Cuneus_L	Temporal_Sup_R	4.227
46	Temporal_Sup_R	Temporal_Pole_Sup_R	4.107
47	Occipital_Inf_R	Temporal_Mid_L	4.552
48	Frontal_Sup_L	Temporal_Mid_R	4.224
49	Frontal_Inf_Orb_R	Temporal_Mid_R	4.779
50	Rolandic_Oper_L	Temporal_Mid_R	4.157
51	Supp_Motor_Area_R	Temporal_Mid_R	4.144
52	Olfactory_R	Temporal_Mid_R	4.395
53	Temporal_Pole_Sup_R	Temporal_Mid_R	4.594
54	Precentral_L	Temporal_Pole_Mid_R	4.218
55	Temporal_Mid_R	Temporal_Pole_Mid_R	4.536
56	Frontal_Mid_Orb_R	Temporal_Inf_L	4.179
57	Precentral_L	Temporal_Inf_R	5.055
58	Frontal_Sup_L	Temporal_Inf_R	4.192
59	Frontal_Sup_Orb_R	Temporal_Inf_R	4.161
60	Frontal_Mid_Orb_R	Temporal_Inf_R	4.739
61	Frontal_Inf_Orb_R	Temporal_Inf_R	5.228
62	Rolandic_Oper_L	Temporal_Inf_R	4.637
63	Olfactory_L	Temporal_Inf_R	4.635
64	Rectus_R	Temporal_Inf_R	4.181
65	Postcentral_L	Temporal_Inf_R	5.068
66	Parietal_Inf_L	Temporal_Inf_R	4.221
67	Temporal_Sup_L	Temporal_Inf_R	4.664
68	Temporal_Mid_L	Temporal_Inf_R	4.290
69	Temporal_Pole_Mid_R	Temporal_Inf_R	4.261
